# Strand-specific community RNA-seq reveals prevalent and dynamic antisense transcription in human gut microbiota

**DOI:** 10.3389/fmicb.2015.00896

**Published:** 2015-09-01

**Authors:** Guanhui Bao, Mingjie Wang, Thomas G. Doak, Yuzhen Ye

**Affiliations:** ^1^School of Informatics and Computing, Indiana UniversityBloomington, IN, USA; ^2^Department of Biology, Indiana UniversityBloomington, IN, USA; ^3^National Center for Genome Analysis Support, Indiana UniversityBloomington, IN, USA

**Keywords:** metatranscriptome, metagenome, antisense RNA, human gut microbiota, transposases

## Abstract

Metagenomics and other meta-omics approaches (including metatranscriptomics) provide insights into the composition and function of microbial communities living in different environments or animal hosts. Metatranscriptomics research provides an unprecedented opportunity to examine gene regulation for many microbial species simultaneously, and more importantly, for the majority that are unculturable microbial species, in their natural environments (or hosts). Current analyses of metatranscriptomic datasets focus on the detection of gene expression levels and the study of the relationship between changes of gene expression and changes of environment. As a demonstration of utilizing metatranscriptomics beyond these common analyses, we developed a computational and statistical procedure to analyze the antisense transcripts in strand-specific metatranscriptomic datasets. Antisense RNAs encoded on the DNA strand opposite a gene’s CDS have the potential to form extensive base-pairing interactions with the corresponding sense RNA, and can have important regulatory functions. Most studies of antisense RNAs in bacteria are rather recent, are mostly based on transcriptome analysis, and have been applied mainly to single bacterial species. Application of our approaches to human gut-associated metatranscriptomic datasets allowed us to survey antisense transcription for a large number of bacterial species associated with human beings. The ratio of protein coding genes with antisense transcription ranges from 0 to 35.8% (median = 10.0%) among 47 species. Our results show that antisense transcription is dynamic, varying between human individuals. Functional enrichment analysis revealed a preference of certain gene functions for antisense transcription, and transposase genes are among the most prominent ones (but we also observed antisense transcription in bacterial house-keeping genes).

## Introduction

Advances in sequencing technology have catalyzed the development of metagenomics, which has revolutionized many fields in the study of microbial organisms. Metagenomics has been applied to study microbial communities sampled from various environments and animal hosts (including humans). Several large-scale efforts worth mentioning are the early global ocean surveys (Nealson and Venter, 2007; Rusch et al., 2007), and more recent MetaHit (Qin et al., 2010) and the NIH Human Microbiome Project (HMP; Human Microbiome Project Consortium, 2012a,b; thanks to which the composition of the human microbiome is now well-studied). The research emphasis now has shifted toward elucidating the functionality and regulatory mechanisms of the microbial communities using other meta-omics approaches, including metatranscriptomics and metaproteomics.

Metatranscriptomics research is creating an unprecedented opportunity to gain knowledge about gene regulation for many microbial species simultaneously, and more importantly, for the vast majority of uncultured microbial species in their natural environments (or hosts). In addition to elucidating functional characteristics of microbial communities, metatranscriptomic data provides information vital for accurate annotations of genes and their regulation in their community—complementary to metagenomic sequencing. Metatranscriptomic data indicate which of the genes encoded in a metagenome are actually transcribed, and which metabolic pathways are active (and the level of their activities), on the basis of their transcripts within a microbial community under various environmental conditions.

Current analyses of metatranscriptomic datasets have largely been limited to the detection of gene expression levels and the relationship between gene expression (and functions and pathways involved) and changes in environmental conditions (de Menezes et al., 2012; Leimena et al., 2013; Franzosa et al., 2014; Jorth et al., 2014; Coolen and Orsi, 2015). However, metatranscriptomics datasets contain rich information, which can be utilized to address important questions, when powered with appropriate computational and statistical approaches. For example, antisense RNAs (asRNAs; Jens and Wolfgang, 2011), which are encoded on the DNA strand opposite to a protein coding (sense) gene transcript (so may play important regulatory roles by forming extensive base-pairing interactions with the corresponding sense RNA), can be revealed by strand-specific metatranscriptomic sequences.

In a standard metatranscriptomic study (using the RNA-seq protocol), total RNA is isolated from the sample, ribosomal RNAs are removed to enrich for mRNA, which is then reverse transcribed into cDNA and subjected to DNA sequencing, using next generation sequencing (NGS) platforms (Giannoukos et al., 2012). It is important to remove the ribosomal RNAs during the process, otherwise the majority of reads from a metatranscriptomic project are rRNA (He et al., 2010). Early metatranscriptomic methods lacked strand specificity, limiting the application of metagenomic datasets in elucidating some regulatory mechanisms in bacteria. However, Giannoukos et al. (2012) presented a protocol for metatranscriptomic analysis of bacterial communities that accommodates both intact and fragmented RNA and combines efficient rRNA removal with strand-specific RNA-seq. Currently, only a handful of such metatranscriptomic datasets are available (and metaproteomic datasets are even scarcer), but we envision a flood of strand-specific RNA-seq metatranscriptomic data in the near future, as experimental techniques mature (Giannoukos et al., 2012; Franzosa et al., 2014).

Antisense RNAs encoded on the DNA strand opposite a gene have the potential to form extensive base-pairing interactions with the corresponding sense RNA (Thomason and Storz, 2010). Unlike other—smaller—regulatory RNAs in bacteria, antisense RNAs range in size from 10 to 1000s of nucleotides, complementary to part of a gene, a complete gene or a group of genes in an operon (Beiter et al., 2009). Although antisense RNAs were first observed in bacteria in the early 1980s (Lacatena and Cesareni, 1981) and their regulatory roles were defined in model systems (Green et al., 1986), most studies of antisense RNAs in bacteria are rather recent. Many antisense RNAs were identified using genome-wide searches for sRNAs and from transcriptome analysis, and have been studied mainly for single bacterial species. The numbers of antisense RNAs reported for different bacteria vary extensively, but 100s have been suggested in some species (Thomason and Storz, 2010). For example, 1,005 antisense RNAs (22% of all genes) were reported for *Escherichia coli* (Dornenburg et al., 2010). Massive antisense transcription was observed for *Synechocystis* PCC6803, with 26.8% of its genes reported to have antisense transcription (Mitschke et al., 2011), and genome-wide antisense transcription was observed in *Helicobacter pylori* (Sharma et al., 2010). Many species have less antisense transcription: for example, only 1.3% of the genes in *Staphylococcus aureus* were reported to have antisense transcription (Beaume et al., 2010). Thomason and Storz (2010) noted in their review that the existence of antisense RNAs was not tested for in many studies.

The availability of human-associated strand-specific metatranscriptomics datasets allows us to examine the antisense transcriptions for a large number of microbial species growing in their natural communities. In this paper we developed computational and statistical approaches to identify antisense transcripts from human gut-associated microbial species and study their dynamics among different human individuals.

## Materials and Methods

### Dataset

We used the human gut-associated strand-specific metatranscriptomic data from (Franzosa et al., 2014); the datasets were downloaded from the SRA website (SRA accession: SRR769395-SRR769540). In total, we analyzed eight sets of metatranscriptomic datasets; each set contains three metatranscriptomic datasets derived from the same human individual, but prepared using three different methods of sample preservation (frozen, ethanol-fixed, or RNAlater-fixed; Franzosa et al., 2014). The eight individuals are X310763260 (abbreviated as X1), X311245214 (X2), X316192082 (X3), X316701492 (X4), X317690558 (X5), X317802115 (X6), X317822438 (X7), and X319146421 (X8).

Bacterial reference genomes (including the genomic sequences and gene annotations) were downloaded from the NCBI ftp site (ftp://ftp.ncbi.nlm.nih.gov/genomes/bacteria/). We focused on 116 reference genomes (covering 47 species), which were reported as the main species found in stool samples (Franzosa et al., 2014). For some analyses, including the function enrichment analysis, we selected a representative strain for each species with multiple strains, to limit the biases that may be introduced by the uneven sampling of the species. See Data Sheet 1 for the list of 116 strains, and the list of 47 representative strains and the basic information about the genome (e.g., the number of genes found in each genome).

### Identification of Sense and Antisense Reads

Raw reads were trimmed with Trimmomatic 0.33 (Bolger et al., 2014) to remove adapter sequences and low quality reads and the trimmed reads were mapped to the 116 bacterial genomes with Bowtie 2 (Langmead and Salzberg, 2012). For simplicity, we call a read that maps to the sense strand a *sense read*, and a read mapped to the antisense strand of a gene an *antisense read*. We used featureCounts twice on the same dataset with the strand setting reversed (-s 1 and then -s 2) to annotate sense and antisense reads (Liao et al., 2014): featureCounts counts mapped reads for genomic features including genes, promoters, gene bodies, and chromosomal locations (given in an input annotation file) and outputs the number of reads assigned to each feature.

We summarize the antisense transcription at both read and gene levels. For each species, we computed two ratios: the *ratio of antisense reads* (out of all reads that can be mapped to the protein coding genes in this species), and the *ratio of genes with antisense transcription* (see below for the detection of genes with antisense transcription using sequencing data).

### Detection of Antisense Expression by a Binomial Test

Artifacts introduced by cDNA synthesis and amplification are known problems for antisense RNA detection (Thomason and Storz, 2010), so even for a gene with no actual antisense transcription, we may find reads suggesting antisense transcripts (i.e., the strandedness of RNA-seq data is <100%). To overcome this problem, we use binomial testing to detect genes with antisense transcripts that are unlikely to be the results of such artifacts: let *p* be the probability of having reads from the antisense strand of a gene, even though there is no real antisense transcription from the gene. A total of *c* reads are sequenced from the gene (*c* is approximated as the number of reads that can be mapped to the gene), among which *m* reads represent antisense transcript. The null hypothesis is that there is no antisense transcription from this gene. We use the binomial test in R (binom.test) to calculate the probability of having *c* antisense reads (the number of successes) out of *m* trials (a total of *m* reads) with a success rate of *p*. If the probability is low (≤ 0.05 according to one-tailed binomial test), we consider that the gene has antisense transcription (the alternative hypothesis).

Since *p* (the success rate) is usually unknown for metatranscriptomic datasets (but it was shown that most library treatments in RNA-seq have a strandedness of >95% Sigurgeirsson et al., 2014), we use the lowest ratio of antisense reads from individual bacterial species present in the microbial communities to approximate the *p* (considering that the strandedness of the RNA-seq will be at least this good). For the human-gut metatranscriptomics datasets we tested, *p* is 0.01. Using this probability of success, we identified significant antisense transcription for different bacterial species using binomial tests. We also checked which species recruited the most RNA-seq reads (to their protein coding genes), as compared to other species in the eight individuals; their ratios of antisense reads are: 0.0233 and 0.0312, for *Methanobrevibacter smithii* ATCC 35061 in X2 (individual 2) and X8, respectively; 0.0481, 0.0626, and 0.0347 for *Parabacteroides distasonis* ATCC 8503 in X1, X4, and X7, respectively; 0.0296 for *Ruminococcus bromii* in X3; and 0.0078 and 0.0167 for *B. vulgatus* ATCC 8482 in X5 and X6, respectively. Seven out of these eight ratios are <5% (two are close to 1%), consistent with the reported strandedness of most stranded library methods in RNA-seq (>95%; Sigurgeirsson et al., 2014). Thus, we believe that 5% (i.e., strandedness of 95%) is a generous estimate of *p* for the data sets we used, and we also used this *p* to provide a more conservative estimate of the genes with antisense transcription in the data sets we analyzed for comparison purposes.

### Functional Enrichment Analysis of Genes with Antisense Expression

Functional enrichment analysis was conducted using two different tests for Clusters of Orthologous Groups (COG; Tatusov et al., 1997). We used the representative set of strains (47 in total) for this analysis, and gene annotations for their genomes were downloaded from the NCBI ftp website.

A one-tailed binomial test with Benjamini-Hochberg (BH) false discovery rate (FDR) correction (*q* ≤ 0.05) was first used to determine if a COG was significantly enriched in the set of genes with antisense expression. The frequency of a COG among all the COGs for a bacterial genome was used as the hypothesized probability of success for the test. In the subset of genes detected to have antisense expression, the number of occurrences of a COG is considered the number of successes, and the total number of detected genes with antisense expression was used as the number of trials. To ensure the binomial test was conducted in a sufficiently large sample, we only tested genomes with ≥ 30 genes with antisense expression. For example, 71 out of 2,204 protein coding genes from *Bacteroides salanitronis* DSM 18170 were detected to have antisense transcription, and 10 out 33 genes that belong to COG4974L were detected to have antisense transcription. Here the number of successes, the number of trials, and the probability of success are 10, 71, and 0.0032 (33/2204), respectively. By the binomial test, the *p*-value was computed to be 1.14e-07, which was then corrected for multiple testing. This resulted in a *q*-value of 6.25e-06, indicating a significant enrichment of COG4974L among genes with antisense expression in this species.

For the enrichment analysis, we noted the binomial test with FDR correction penalized heavily for COGs with few genes. Therefore, we also investigated the association between COG family and antisense expression by a one-tailed Fisher’s exact test with BH FDR correction (*q* ≤ 0.05). For the example above (*B. salanitronis* DSM 18170), the 2 by 2 contingency table is [(10, 23), (61, 2110)] and the *q*-value was calculated to be 1.78e-06, also indicating the enrichment of COG4974L in genes with antisense transcription in the genome.

## Results

### Sample Preservation Method Matters for the Detection of Antisense Transcription from Metatranscriptomic Sequences

Franzosa et al. (2014) used three different methods for preserving samples (frozen, ethanol-fixed, or RNAlater-fixed) for metatranscriptomics sequencing. They showed that measurements of microbial species, gene, and gene transcript composition within self-collected samples were consistent across sampling methods (Franzosa et al., 2014). We first asked if this consistency applied to antisense transcription.

**FIGURE 1:**
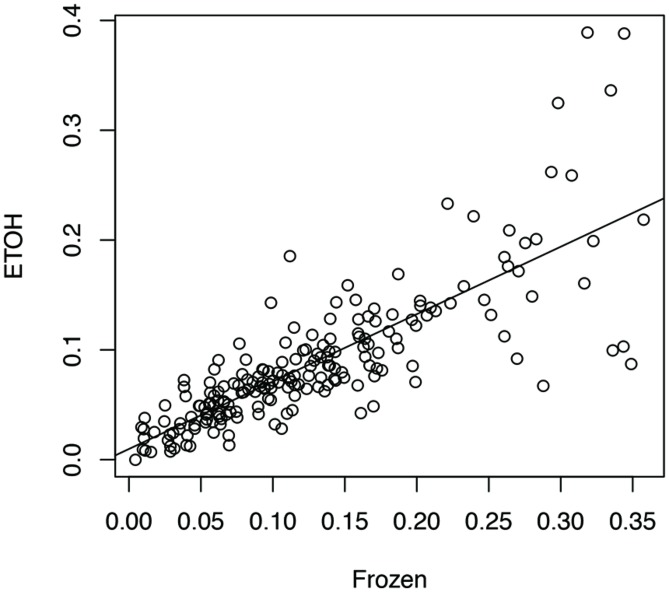
**Impacts of different experimental protocols on the profiling of antisense transcription.** The figure shows the correlation (and differences) between the profile of antisense transcription (i.e., ratios of genes with antisense transcription in different species across individuals) for frozen samples and the profile of antisense transcription for ethanol-fixed samples (Pearson’s *r* = 0.71; two tailed *p*-value < 2.2e-16). In this plot, the ratios of genes with antisense transcription for the different species based on the frozen samples (Frozen) and ethanol-fixed samples (ETOH) are plotted in the x-axis and y-axis, respectively.

We aligned the eight sets of stool metatranscriptome data against the bacterial reference genomes reported as the main species found in stool samples (Franzosa et al., 2014). For each sample handling method, we computed a profile of antisense transcription, in which a number represents the ratio of genes with antisense transcription in one species in one human individual. To limit the bias that may be introduced by uneven sampling of the strains and species with few RNA-seq reads, we only used one strain for each species, and only kept the ratios calculated for species with at least 100 genes supported by RNA-seq reads in an individual prepared by all three experimental methods (see Human Gut-Associated Microbial Organisms have a Wide Range of Antisense Transcription). In total 196 ratios for each handling method were included for the analysis. Our results show that all three sample-handling approaches result in highly correlated profiles of antisense transcription, with the frozen samples and the RNAlater-fixed samples sharing the most similar profiles (Pearson’s *r* = 0.84; two tailed *p*-value < 2.2e-16) and RNAlater-fixed samples and ethanol-fixed samples sharing the least similarity (Pearson’s *r* = 0.71; two tailed *p*-value < 2.2e-16). However, differences in the profiles are also obvious, as shown in the comparison between the profiles from ethanol-fixed samples and frozen samples (**Figure 1**).

A two-way ANOVA test of antisense transcriptions of all eight individuals by the three different experimental approaches showed that the handling method has the strongest effect on the antisense transcription (*F* = 7.05, *p*-value = 0.001), followed by the individuals (*F* = 2.88, *p*-value = 0.007), and the interaction between handling methods and individuals (*F* = 1.89, *p*-value = 0.03). A Turkey HSD test further revealed significant differences between frozen samples and ethanol-fixed (*p*-value = 0.0043), and between RNAlater-fixed samples and ethanol-fixed (*p*-value = 0.0068; but not between frozen and ethanol-fixed samples). This result suggests that we be cautious with results based on metatranscriptomic datasets derived from differently preserved samples (although high correlations were observed among these different approaches as shown in **Figure 1**). In addition, the previous publication reported that ethanol-fixed and RNAlater-fixed approaches can cause some artifacts in some functional genes (Franzosa et al., 2014). Considering both, we used the metatranscriptomics datasets generated from frozen samples for all our below analyses.

### Human Gut-Associated Microbial Organisms have a Wide Range of Antisense Transcription

We detected antisense transcription for most of the species we tested. For each species, we computed the ratio of antisense reads (over total reads mapped to the species) and the ratio of genes with antisense transcript (over all genes with detectable transcription; see Materials and Methods). We used datasets derived from all eight individuals (and the ratios for the same species are most likely different in different datasets). The ratios of antisense reads and genes with antisense transcription for all the 116 bacterial strains (covering 47 species) across the samples (from eight individuals), along with other details (such as the total number of mapped reads, antisense reads, etc.), are listed in Data Sheets 1 and 2 in the Supplementary Material.

**FIGURE 2:**
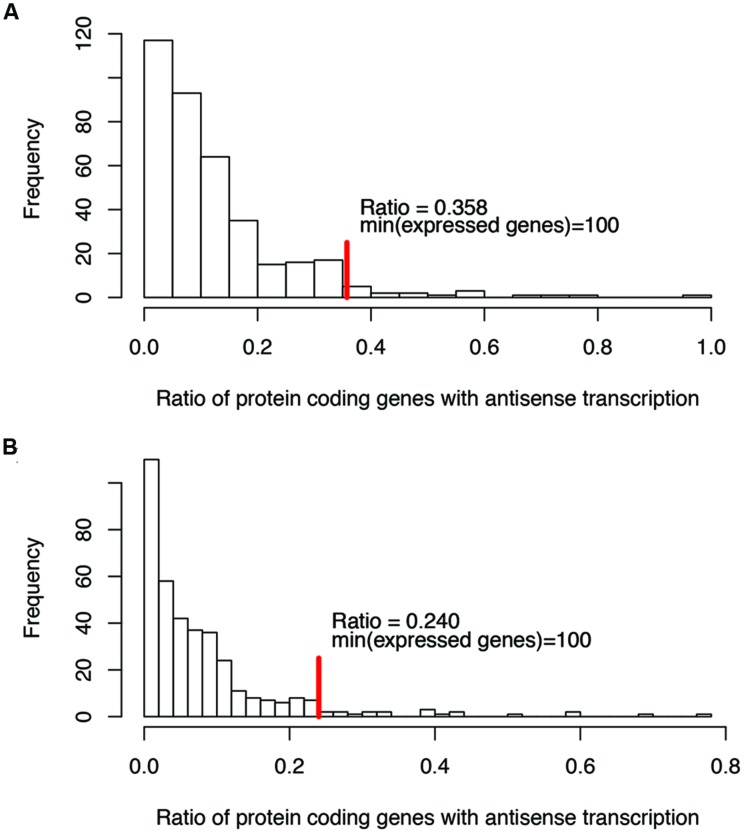
**Histograms of the ratios of genes with antisense transcription (over all genes with detectable transcription).** Binomial tests were used to determine if a gene has antisense transcription or not, with a success rate of 1% **(A)**, and 5% **(B)**, respectively. The red vertical lines indicate the maximum ratios of genes with antisense transcription, for the genomes in which at least 100 genes have detectable transcription (with RNA-seq reads support).

For ratios of genes with antisense transcription, we noticed that some species have extremely high ratios (see the long tails in **Figure 2**; we only considered one strain for each species to reduce the bias that may be introduced by multiple strains belonging to the same species for the histograms), and without exception, all these species have few expressed genes (e.g., with <100 of their genes having detectable transcription). Considering that species with few supporting RNA-seq reads tend to be influenced heavily by potential artifacts (due to ambiguous reads mapping, bad gene annotations, etc.), we only considered species with at least 100 of their genes supported by RNA-seq reads, to infer the range of genes with antisense transcription. The ratio of protein coding genes with antisense transcription ranges from 0 to 35.8% (median = 10.0%; **Figure 2A**), based on the binomial tests using a success rate of 1%; the range drops, to between 0 and 24.0% (median = 6.3%; **Figure 2B**) when the more generous estimate of the success rate (5%, indicating a 95% strandedness of the RNA-seq experiments) was used for the binomial testing. In the following, results are based on binomial testing using *p* of 1%, unless stated otherwise.

**FIGURE 3:**
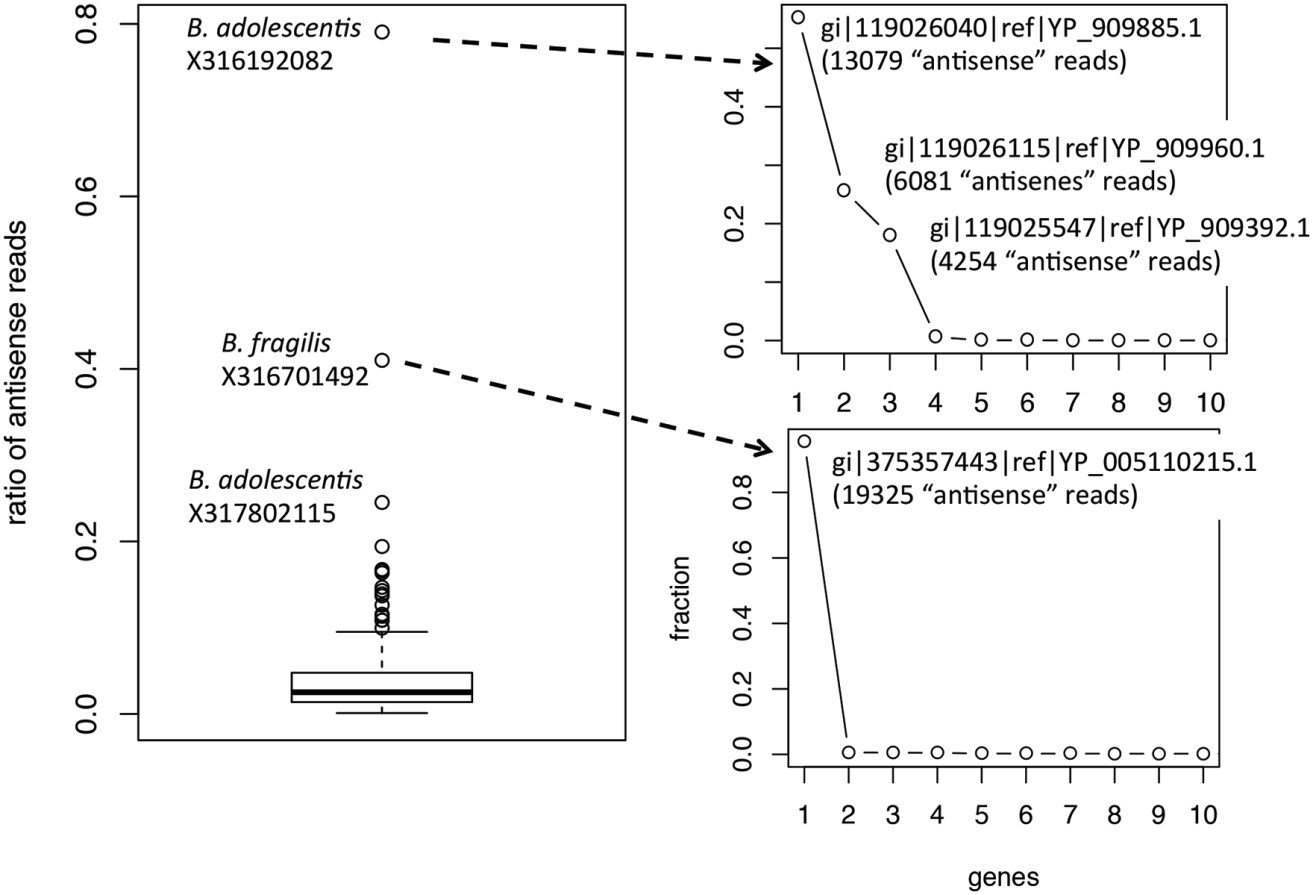
**Boxplot of the ratios of antisense reads (left).** The plots on the right show the contribution of individual genes to the total antisense reads for the two outliers *B. adolescentis* and *B. fragilis*. Most of the antisense reads came from three and one genes (likely misannotations) for *B. adolescentis* and *B. fragilis*, respectively.

Ratios of antisense reads (over total reads mapped to protein coding genes) are generally smaller than ratios of genes with antisense transcription. Similar to the inference of ratios of genes, only species with at least 100 of their genes supported by RNA-seq reads in a dataset were used to infer the range of ratios of antisense reads. **Figure 3** shows the boxplot for the ratios of reads mapped to the antisense strands of protein coding genes: the 95% confidence interval is 0.35–16.3% and the median is 2.5%. The boxplot revealed a few ratios that are significantly higher than the remaining: including the ratio for *B. adolescentis* in individual X316192082, the ratio for *B. fragilis* in individual X316701492, and the ratio for *B. adolescentis* in individual X317802115. As shown in **Figure 3**, for these outliers, most of the “antisense” reads are from a few putative genes (three genes in *B. adolescentis*; and one in *B. fragilis*) that have recruited large numbers of RNA-seq reads; all are hypothetical protein coding genes encoding small proteins without detailed functional annotation (except for gene gi| 119026115|ref| YP_909960.1 in *B. adolescentis*, which was annotated as a DEAD helicase in NCBI annotation; however, searching this protein against the Pfam database revealed no hits). We suspect that these genes are likely ncRNA genes, instead of protein coding genes, and therefore these few large ratios of antisense reads need to be interpreted with caution.

**FIGURE 4:**
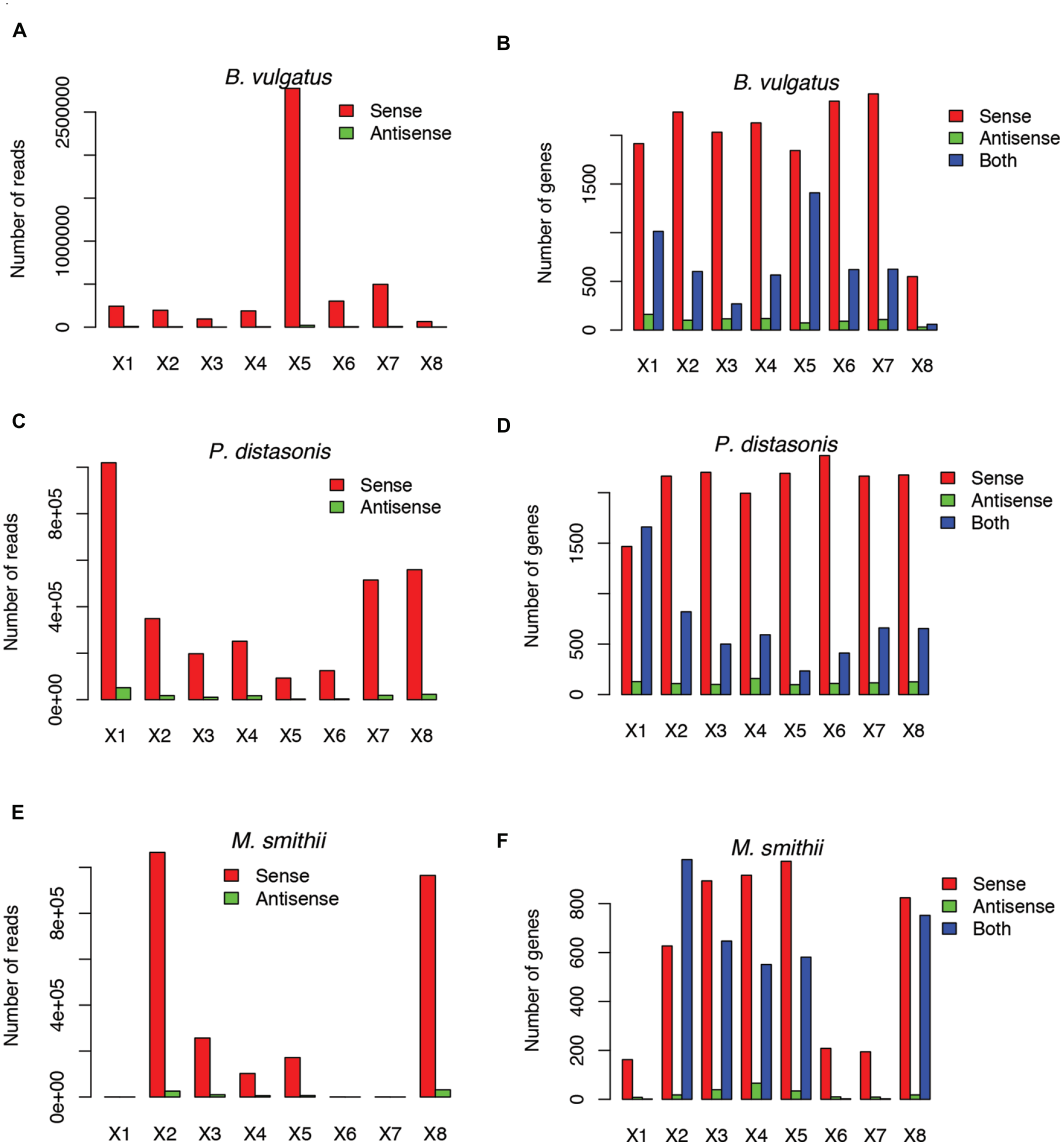
**Example species with antisense transcription in different individuals.** Three species are shown: *Bacteroides vulgatus* ATCC 8482 **(A,B)**, *Parabacteroides distasonis* ATCC 8503 **(C,D)** and *Methanobrevibacter smithii* ATCC 35061 **(E,F)**. **(A,C,E)** Shows the numbers of sense and antisense reads in these three species, and **(B,D,F)** show the number of genes with sense transcription only (Sense), antisense transcription only (Antisense), and both (Both). X1–X8 indicate the eight individuals.

**FIGURE 5:**
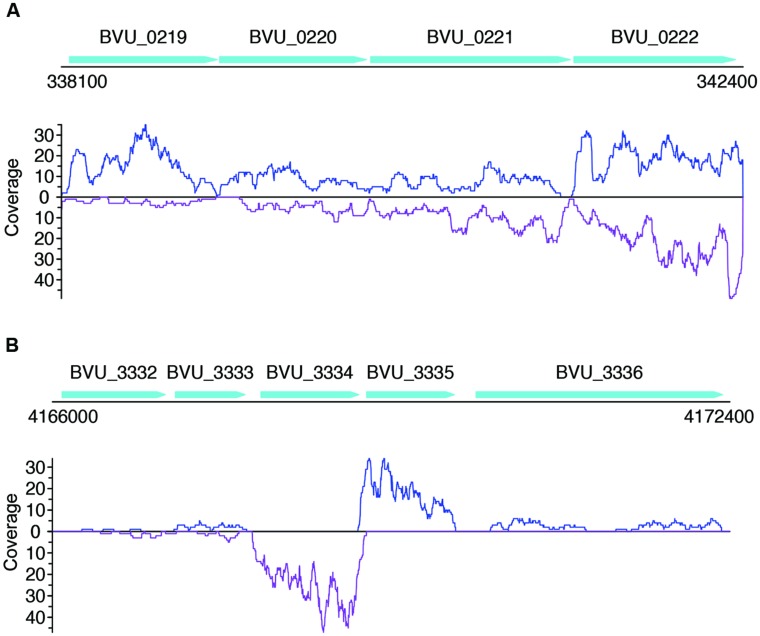
**Read coverage plots for example genes in *B. vulgatus*.** Genes are represented as arrows in the plots, and the read coverage curves are shown below the genes, with the coverage for sense and antisense reads shown in blue and purple, respectively. **(A)** Read coverage plot for an operon with four genes, shown as cyan arrows on the top: BVU_0219 is a putative aldo/keto reductase, BVU_0220 is a hypothetical protein, BVU_0221 is a putative fucose permease, and BVU_0222 is a putative sorbitol dehydrogenase. **(B)** Read coverage plot for BVU_3334 (and its neighboring genes): BVU_3334 is a putative transcriptional regulator, BVU_3333 is similar to a fructose-6-phosphate aldolase from *E. coli*, BVU_3332 is a putative ABC transporter permease, BVU_3335 is a hypotentical protein, and BVU_3336 is a putative glycosyl transferase.

Not surprisingly, most of the strains we tested recruited many more sense than antisense reads, and tend to have more genes with sense transcription than genes with antisense transcription (such as *B. vulgatus* ATCC 8482, as shown in **Figures 4A,B**, *Parabacteroides distasonis* ATCC 8503 as shown in **Figures 4C,D**, and *M. smithii* ATCC 35061 as shown in **Figures 4E,F**). Our results are consistent with a previous study (Franzosa et al., 2014), showing that *M. smithii* is abundant and highly transcriptionally active (supported by huge numbers of RNA-seq reads) in five of the eight individuals (**Figures 4E,F**). But for these species, individual genes may still have significant antisense transcription or even have antisense transcription only; for examples, **Figure 5A** shows the read coverage plot for an operon in *B. vulgatus* (the operon information was extracted from the Database of Prokaryotic Operons; Mao et al., 2009), showing that all four genes in this operon have both sense and antisense transcription; and **Figure 5B** shows that gene BVU_3334 (which encodes for a putative transcriptional regulator) only has antisense reads.

**FIGURE 6:**
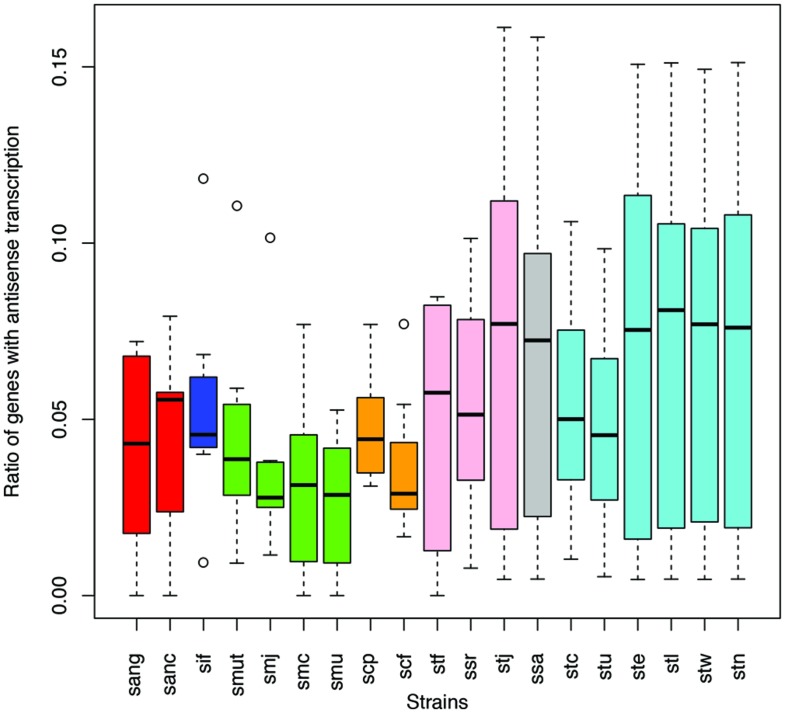
**Different *Streptococcus* species have different levels of antisense transcripts.** The y-axis shows the ratio of genes with antisense transcription. The x-axis shows the different species; sang: *S. anginosus* C1051, sanc: *S. anginosus* C238, sif: *S. infantarius* CJ18, smut: *S. mutans* GS5, smj: *S. mutans* LJ23, smc: *S. mutans* NN2025, smu: *S. mutans* UA159, scp: *S. parasanguinis* ATCC 15912, scf: *S. parasanguinis* FW213, stf: *S. salivarius* 57 I, ssr: *S. salivarius* CCHSS3, stj: *S. salivarius* JIM8777, ssa: *S. sanguinis* SK36, stc: *S. thermophilus* CNRZ1066, stu: *S. thermophilus* JIM 8232, ste: *S. thermophilus* LMD 9, stl: *S. thermophilus* LMG 18311, stw: *S. thermophilus* MN ZLW 002, stn: *S. thermophilus* ND03. The boxplots for the different strains of the same species are shown in the same color.

Different species of the same genus showed various ratios of antisense transcripts. **Figure 6** shows the ratio of genes with antisense transcription in different species of *Streptococcus* (one of the dominant genera in human gut microbiota) across the eight human individuals. Overall, *Streptococcus* species have relatively low antisense transcription: the median of the ratios of antisense reads is 1.1% and the median of the ratios of genes with antisense transcription is 4.4%. *S. mutans* and *S. parasanguinis* have the lowest ratio of genes with antisense transcription; other *Staphylococcus* species seem to have higher antisense transcription, but the ratios vary greatly across different individuals. Similar trends are observed in a plot that shows the ratios of antisense reads for these species (Supplementary Figure S1).

### Genes with either Sense- or Antisense-Dominating Transcription are Typically Highly Expressed

**FIGURE 7:**
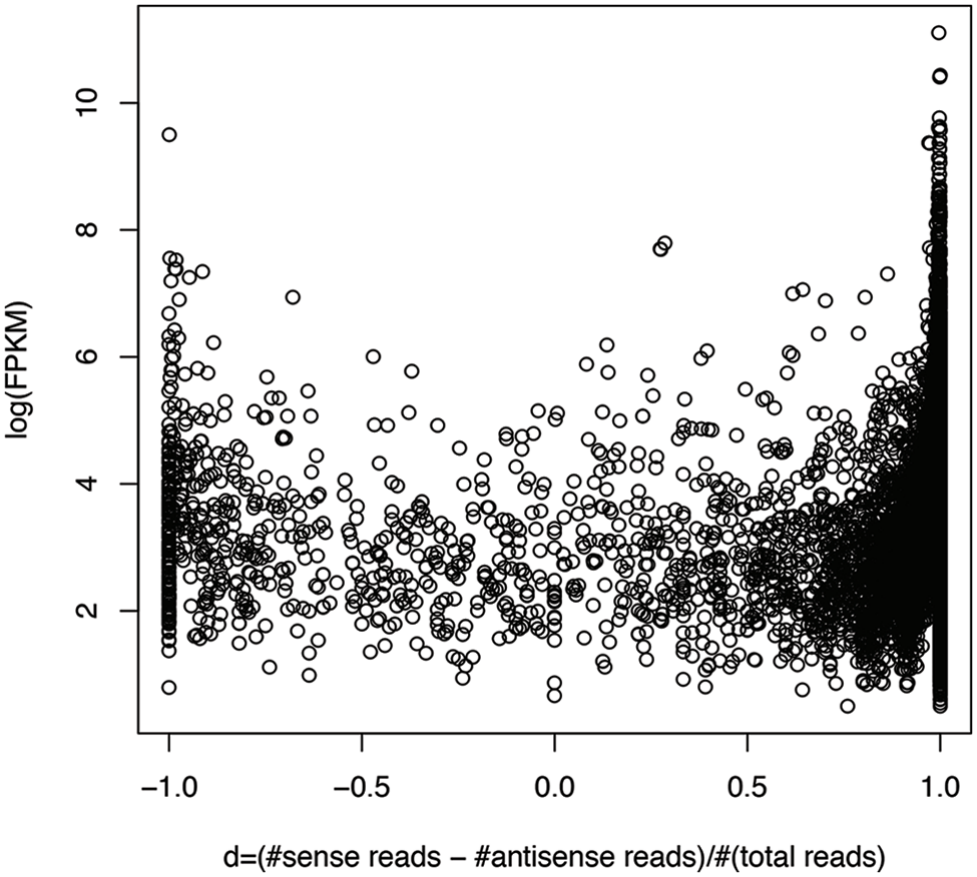
**Highly expressed genes tend to be dominated by sense transcription or antisense transcription.** Each circle represents a gene. The y-axis shows the gene expression (*log*(FPKM)) and the x-axis shows *d*, which is close to 1 for genes with mostly sense transcription, and -1 with mostly antisense transcription. RNA-seq data from individual 1 (X310763260) was used for this plot. To limit the bias that may be introduced by rare species or genes with low expression levels, we only included the genes (3,689 in total) each supported by at least 20 RNA-seq reads, and are from the species (23 in total) each having at least 100 genes with RNA-seq reads support. See Supplementary Figure S2 for the plot using the original gene expression data, involving 30,493 genes from all 47 strains, one for each species.

We can roughly group genes into three categories: genes with mostly sense transcripts, genes with mostly antisense transcripts, and genes in between, based on their sense and antisense transcription. We define *d* = (#sense reads – #antisense reads)/(#sense + #antisense reads), so that genes with mostly sense transcripts have *d* that is close to 1, while genes with mostly antisense transcripts have *d* that is close to -1. **Figure 7** shows the plot of gene expression levels versus the d ratios, using expressed genes from 23 species (each having at least 100 genes with detectable expression), based on the RNA-seq dataset of individual 1 (X310763260; see Supplementary Figure S2 for the plot using all 47 strain; only one strain was included for each species). We used FPKM (Fragments Per Kilobase of transcript per Million mapped reads; Garber et al., 2011) to quantify the gene expression levels, to normalize read counts by the gene length and sequencing depth of the RNA-seq experiments. The number of mapped reads for a dataset was computed as the total number of reads that can be mapped to one of the 116 strains. The plot reveals a “U” shape, indicating that genes with either sense- or antisense-dominated transcription are typically highly expressed, while genes in between have relatively low gene expression. This correlation is confirmed by a statistical test: the Spearman’s correlation coefficient between log(FPKM) and |d| for the genes (each recruited at least 20 RNA-seq reads) shown in **Figure 7** (excluding the genes with d ratios of 1 or -1) is 0.57 (*p*-value < 2.2e-16). Similar results can be observed using an unfiltered dataset from this individual (Spearman’s *r* = 0.69, *p*-value < 2.2e-16), and RNA-seq datasets from other individuals.

We note that a large fraction of genes have either sense transcription only (which is not surprising), or antisense transcription only. For example, for the dataset X310763260 used in **Figure 7** and Supplementary Figure S2, a total of 6,119 protein coding genes (out of 30,493; 20.1%) have antisense transcription according to the binomial testing (success rate = 1%); among which, 1,877 genes only have antisense transcription. We expect this large ratio (1,877/6,119 = 30.7%) of genes with antisense transcription can be only partially contributed by bad gene annotations (which, however, will be difficult to quantitatively estimate without further experimental proofs). But there are still 430 genes if we only included genes at least 600 bp long (longer genes are more likely to be correctly predicted), with at least three RNA-seq reads mapped to their antisense strands (but no reads mapped to sense strands). The gene BVU_3334 in *B. vulgatus* ATCC 8482 mentioned above (**Figure 5B**) is one of such genes: a total of 257 reads were mapped to its antisense strand, but none to the sense strand.

### Dynamic Antisense Transcription in Human Population

**FIGURE 8:**
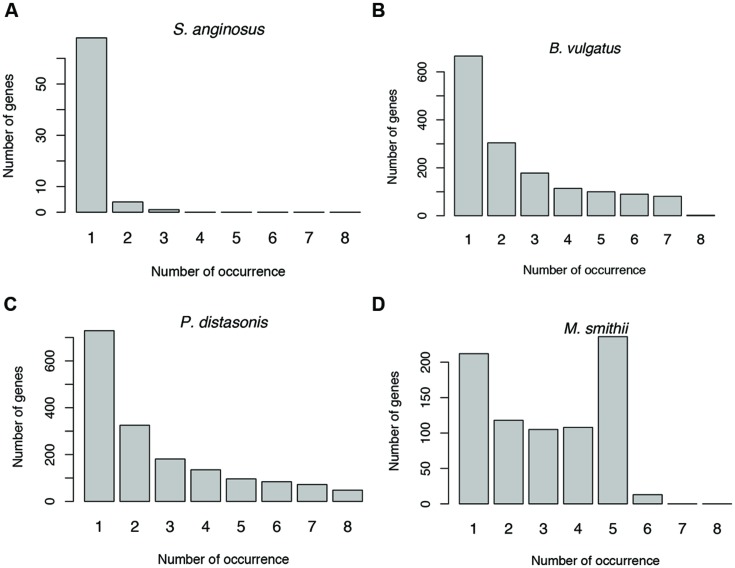
**Sharing of genes with antisense transcription among human individuals.** Genes associated with *Streptococcus anginosus* C238 **(A)**, *Bacteroides vulgatus* ATCC 8482 **(B)** and *Parabacteroides distasonis* ATCC 8503 **(C)** tend to be unique to different individuals, while genes associated with *Methanobrevibacter smithii* ATCC 35061 tend to be shared by individuals **(D)**. The numbers below the bars indicate the number of individuals sharing the genes with antisense transcription, with 1 indicating the number of genes unique to one individual, and 2–8 for genes shared by two individuals, and then increasing numbers of individuals.

Antisense transcription varies between human individuals. For example, as shown in **Figure 6** (and Supplementary Figure S1), the prevalence of antisense transcription in different *Streptococcus* species varies across human individuals. In addition, the actual genes that have antisense transcripts vary greatly: most of the genes with antisense transcription are only found to have antisense transcription in one or only a few individuals (**Figures 8A–C**). For example, a total of 1,535 protein coding genes (out of 4,067; 38%) in *B. vulgatus* ATCC 8482 are found to have antisense transcription in at least one of the eight individuals; however, only two genes are common in all individuals, while 666 genes are found in only one of the individuals (**Figure 8B**). *M. smithii* ATCC 35061 is an exception (**Figure 8D**): many of its genes with antisense transcription are common among the individuals. A total of 792 protein coding genes (out of 1,793 genes; 44%) are found to have antisense reads in at least one of the eight individuals, and 236 of these genes have antisense reads in five individuals (note that *M. smithii* was found to be abundant only in five out of the eight individuals; see **Figures 4E,F**).

### Functions Enriched in Genes with Antisense Transcription

**FIGURE 9:**
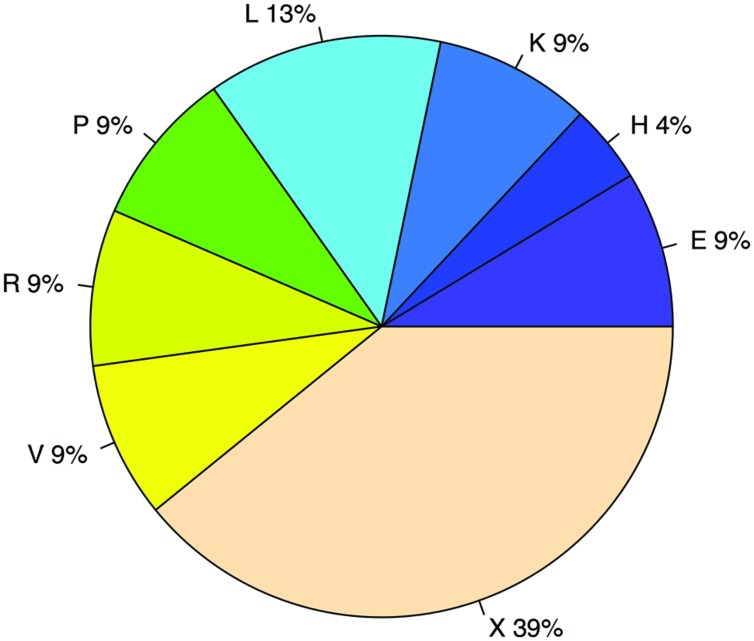
**COG functional categories enriched in genes with antisense transcription.** The functional categories include E: Amino acid transport and metabolism; H: Coenzyme transport and metabolism; K: Transcription; L: Replication, recombination and repair; P: Inorganic ion transport and metabolism; R: General function prediction only; V: Defense mechanisms; and X: Mobilome, prophages, transposons.

We used two different statistical tests to detect if genes encoding certain functions tend to have antisense transcription: **Table 1** lists the COG functions that are enriched in genes with antisense transcription based on the Fisher’s exact test with BH FDR correction. The binomial tests gave consistent results but with fewer COGs detected to be enriched (see Supplementary Table S1 for details). **Figure 9** summarizes the COG functional categories enriched in the genes (associated with the 47 species we tested; only one strain was selected for each species) that have observed antisense transcription. The most significant category is X (mobilome, prophages, and transposons), which has eight COG functions that are significantly enriched in genes with antisense transcription. The next category L, replication, recombination and repair, contains two enriched COG functions (COG1961 and COG4974). Transposases are among the genes frequently identified to have antisense transcription in previous studies: RNA-OUT of the transposon Tn10 (one of the first discovered antisense RNAs), was found to repress transposition by reducing transposase levels (Simons and Kleckner, 1983); and in a study of non-coding RNAs in the archaeon *Sulfolobus solfataricus* (Tang et al., 2005), the most prominent group of antisense RNAs was found to be transcribed in the opposite orientation to the transposase genes encoded by insertion elements (the authors of the paper hypothesized that these antisense RNAs regulate transposition of insertion elements by inhibiting expression of the transposase mRNA). We also identified other functions that are enriched in genes with antisense transcription, which may provide clues to the regulation of these genes.

**Table 1:** Clusters of Orthologous Groups (COG) functions that are enriched in the genes with antisense transcription in 47 strains (*q*-value < = 0.05 by Fisher’s exact test with FDR correction).

COG ID	Cat^$^	Strains^#^	Function description	*q*-value^∗^
COG3842	E	1	ABC-type Fe^3+^/spermidine/putrescine transport systems, ATPase components	0.032
COG0493	E, R	1	NADPH-dependent glutamate synthase beta chain or related oxidoreductase	0.032
COG2226	H	1	Ubiquinone/menaquinone biosynthesis C-methylase UbiE	0.029
COG0568	K	1	DNA-directed RNA polymerase	0.0099
COG0583	K	1	DNA-binding transcriptional regulator, LysR family	0.018
COG1961	L	1	Site-specific DNA recombinase related to the DNA invertase Pin	0.033
COG4974	L	2	Site-specific recombinase XerD	2.09e-06; 0.032
COG1178	P	1	ABC-type Fe^3+^ transport system, permease component	0.032
COG2059	P	1	Chromate transport protein ChrA	0.032
COG0628	R	1	Predicted PurR-regulated permease PerM	0.032
COG0534	V	2	Na^+^-driven multidrug eﬄux pump	0.014; 0.018
COG2801	X	1	Transposase InsO and inactivated derivatives	0.018
COG2826	X	2	Transposase and inactivated derivatives, IS30 family	7.57e-07; 0.012
COG3293	X	1	Transposase	0.0068
COG3328	X	1	Transposase (or an inactivated derivative)	0.012
COG3378	X	1	Phage- or plasmid-associated DNA primase	0.029
COG3415	X	1	Transposase	0.029
COG3464	X	1	Transposase	0.029
COG3666	X	1	Transposase	0.042

*^$^Functional categories (check the caption in **Figure 9** for the description of the categories); ^#^Number of strains with detected antisense expression for the corresponding function; ^∗^All q-values will be listed if a function is detected to be enriched in multiple species.*

## Discussion

Strand-specific RNA-seq is a powerful tool for transcript discovery, genome annotation and expression profiling (Levin et al., 2010). In eukaryotes, 1000s of RNAs antisense to protein-coding genes have been revealed via high-throughput sequencing analyses (Berretta and Morillon, 2009). In contrast, few reports have identified antisense to protein-coding genes in bacteria, but previous studies have demonstrated that antisense RNAs can regulate expression of their corresponding genes in bacteria (Brantl, 2007). Although several studies have shown that antisense transcription may be widespread in bacteria, a global analysis of antisense transcripts using strand-specific information has only been reported for several model, cultured strains (Passalacqua et al., 2012; Behrens et al., 2014; Siegel et al., 2014). We describe a computational and statistical procedure to derive antisense transcripts from metatranscriptome data of microbial communities. With this method, we survey the antisense RNAs on a much broader scale than conventional methods, which have focused on single species.

Due to the fact that the strandedness is not 100% for RNA-seq experiments, it is necessary to have a way to correct for the artifacts. We proposed to use a binomial test to determine if a gene is likely to have antisense transcriptions, or the antisense reads are more likely artifacts. It helped to remove some of the artifacts. However, we note that this approach will underestimate the ratio of genes with antisense transcription for the species with few RNA-seq reads. This also indicates that when we compare the ratios of genes with antisense transcription for different species, we need to be cautious about the interpretation in comparing the results.

Mapping reads to bacterial genomes has been difficult due to the existence of closely related species in a microbial community and the limited availability of reference genomes (so the actual species might not be presented by the reference genomes; Wang et al., 2012). We acknowledge there is a potential problem with the assignment of sequencing reads to individual genomes due to the ambiguity of mapping. However, the conclusion we drew based on genes should be robust (the sense strand of a gene in one species is likely to be the sense strand as well for its homologs in related species). Also analysis at the pan-genome level or even genus level may be worth pursuing in the future, which may provide insights into the antisense transcription from different angles.

We note that there are other artifacts that may also have impacts on the analysis of antisense transcriptions and the interpretation of the results. For examples, genomic-DNA contamination may result in the detection of artificial antisense transcriptions (Haas et al., 2012). The different genome sizes for the species in a community, and different gene lengths will complicate the analysis of gene expression (Garber et al., 2011). Gene annotations for most of the genomes contain mistakes, and there are complicated gene structures (such as overlapping genes) that are difficult to be considered for antisense transcription analysis. Finally, for metatranscriptomic studies, the RNA-seq data reflects the compound output of the gene expression and the species abundance, making the interpretation of the results less straightforward.

Antisense transcription can be important for the regulation of some functions, such as transposase genes. One interesting example is the *Bacteroides uniformis* mobilizable transposon NBU1. All of its 10 genes have antisense expression in one individual, and in other individuals also have higher antisense expression for this strain. This result suggests that in most individuals, the inactivation of this transposon by antisense RNAs serves an important regulatory role for its transposition. A further observation is that for a given bacterial species, the set of genes with antisense transcripts varies between human host, suggesting that environmental differences between hosts is leading to antisense-dependent regulatory responses by the resident bacteria.

Genes that have exclusively antisense transcripts are clearly “off”; depending on the efficacy of antisense suppression of sense translation, all genes with >50% antisense may be turned off, for example. And it is not surprising that many genes in a genome are turned off under a given set of conditions. A gene that is repressed for sense expression will naturally show a higher level of antisense expression, even if this is background noise. The question then becomes: are the antisense transcripts we observed actually a mechanism to specifically suppress expression, especially for genes with the highest levels of antisense expression. Strong antisense transcription was detected for the *opa* genes coding for adhesins and invasins, which may have regulatory functions in pathogenic *Neisseria* (Remmele et al., 2014). In the case of transposons, we know that antisense transcripts are a specific mechanism to maintain very low, or episodic, expression (Brantl, 2007). If at least some genes are being regulated by their antisense transcripts, it is no surprise that the levels will vary between different environments, i.e., individuals.

## Author Contributions

GB and MW carried out the analysis and drafted the manuscript. TD participated in the analysis and helped to draft the manuscript. YY conceived the study, participated in its design and coordination, participated in the analysis, and helped to draft the manuscript. All authors read and approved the final manuscript.

## Acknowledgment

This work was supported by NIH grant R01AI108888 and NSF grant DBI-0845685.

## Conflict of Interest Statement

The authors declare that the research was conducted in the absence of any commercial or financial relationships that could be construed as a potential conflict of interest.

## Supplementary material

Data Sheet 1 **| An Excel file with three spreadsheets listing the species included in the analyses, and details of their ratios of antisense reads and genes with antisense transcription in different individuals**.

Data Sheet 2 **| An Excel file with eight spreadsheets listing detailed information about antisense and sense reads for individual protein coding genes in different genomes, across eight samples**.
