# Supplementary material for: Strand-specific community RNA-seq reveals prevalent and dynamic antisense transcription in human gut microbiota
Source: Front Microbiol. 2015 Sep 1;6:896. doi: 10.3389/fmicb.2015.00896 (PMC4555090; doi:10.3389/fmicb.2015.00896)
Supplement: Supplementary file 3 [file Table_1.DOCX]

**This document contains Supplementary Table 1.**

**Supplementary Table 1**. COG functions that are enriched in the genes with antisense transcription (*p*-value < 0.01 by binomial test).

| COG ID | Cat^$^ | Strains^#^ | Function description | *p*-value^*^ |
| --- | --- | --- | --- | --- |
| COG3842 | E | 1 | ABC-type Fe3+/spermidine/putrescine transport systems, ATPase components | 0.0031 |
| COG0493 | E, R | 1 | NADPH-dependent glutamate synthase beta chain or related oxidoreductase | 0.0031 |
| COG2226 | H | 1 | Ubiquinone/menaquinone biosynthesis C-methylase UbiE | 0.0099 |
| COG0596 | H, R | 1 | Pimeloyl-ACP methyl ester carboxylesterase | 0.0067 |
| COG1597 | I, R | 1 | Diacylglycerol kinase family enzyme | 0.0064 |
| COG0050 | J | 1 | Translation elongation factor EF-Tu, a GTPase | 0.0073 |
| COG0568 | K | 1 | DNA-directed RNA polymerase | 0.0007 |
| COG0583 | K | 1 | DNA-binding transcriptional regulator, LysR family | 0.0047 |
| COG1609 | K | 1 | DNA-binding transcriptional regulator, Lacl/PurR family | 0.009 |
| COG3279 | K, T | 1 | DNA-binding response regulator, lytR/AlgR family | 0.0092 |
| COG0210 | L | 1 | Superfamily I DNA or RNA helicase | 0.0067 |
| COG0513 | L | 1 | Superfamily II DNA and RNA helicase | 0.0045 |
| COG1484 | L | 1 | DNA replication protein DnaC | 0.003 |
| COG1961 | L | 1 | Site-specific DNA recombinase related to the DNA invertase Pin | 0.003 |
| COG4974 | L | 3 | Site-specific recombinase XerD | 1.3e-07; 0.0053; 0.0033 |
| COG3772 | M | 1 | Phage-related lysozyme (muramidase), GH24 family | 0.0077 |
| COG1178 | P | 1 | ABC-type Fe3+ transport system, permease component | 0.0031 |
| COG2059 | P | 2 | Chromate transport protein ChrA | 0.0031; 0.0094 |
| COG0628 | R | 1 | Predicted PurR-regulated permease PerM | 0.0019 |
| COG0534 | V | 3 | Na+-driven multidrug efflux pump | 0.0008; 0.0029; 0.0021 |
| COG2801 | X | 1 | Transposase InsO and inactivated derivatives | 0.0026 |
| COG2826 | X | 3 | Transposase and inactivated derivatives, IS30 family | 6.09e-08; 0.0078; 0.0005 |
| COG3293 | X | 1 | Transposase | 0.0007 |
| COG3328 | X | 3 | Transposase (or an inactivated derivative) | 0.001; 0.0094; 0.0044 |
| COG3344 | X | 1 | Retron-type reverse transcriptase | 0.0065 |
| COG3378 | X | 1 | Phage- or plasmid-associated DNA primase | 0.002 |
| COG3415 | X | 1 | Transposase | 0.0057 |
| COG3666 | X | 1 | Transposase | 0.0063 |

The underlined COGs passed the binomial test with Benjamini-Hochberg false discovery rate correction (*q*-value <= 0.05).

^$^: The functional categories include E: Amino acid transport and metabolism; H: Coenzyme transport and metabolism; I: Lipid transport and metabolism; J: Translation, ribosomal structure and biogenesis; K: Transcription; L: Replication, recombination and repair; M: Cell wall/membrane/envelope biogenesis; P: Inorganic ion transport and metabolism; R: General function prediction only; T: Signal transduction mechanisms; V: Defense mechanisms; and X: Mobilome, prophages, transposons;

^#^: Number of strains with detected antisense expression for the corresponding function;

^*^: All *p*-values will be listed if a function is detected to be enriched in multiple species.
