# Supplementary material for: Strand-specific community RNA-seq reveals prevalent and dynamic antisense transcription in human gut microbiota
Source: Front Microbiol. 2015 Sep 1;6:896. doi: 10.3389/fmicb.2015.00896 (PMC4555090; doi:10.3389/fmicb.2015.00896)
Supplement: Supplementary file 4 [file Image_1.PDF]

This document contains Supplementary Figure 1.

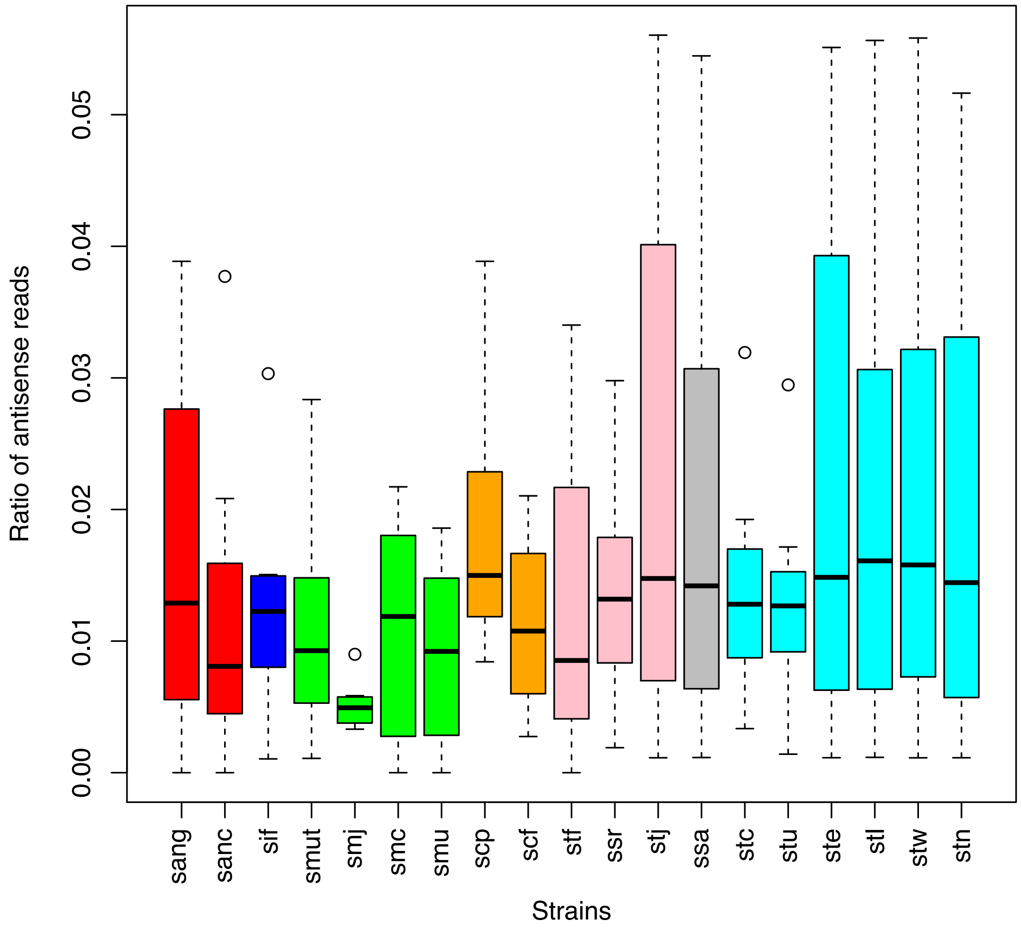

Supplementary Figure 1. Different *Strepcococcus* species have different levels of antisense transcripts. The y-axis shows the ratio of antisense reads. The x-axis shows the different species; sang: *Streptococcus anginosus* C1051, sanc: *Streptococcus anginosus* C238, sif: *Streptococcus infantarius* CJ18, smut: *Streptococcus mutans* GS5, smj: *Streptococcus mutans* LJ23, smc: *Streptococcus mutans* NN2025, smu: *Streptococcus mutans* UA159, scp: *Streptococcus parasanguinis* ATCC 15912, scf: *Streptococcus parasanguinis* FW213, stf: *Streptococcus salivarius* 57 I, ssr: *Streptococcus salivarius* CCHSS3, stj: *Streptococcus salivarius* JIM8777, ssa: *Streptococcus sanguinis* SK36, stc: *Streptococcus thermophilus* CNRZ1066, stu: *Streptococcus thermophilus* JIM 8232, ste: *Streptococcus thermophilus* LMD 9, stl: *Streptococcus thermophilus* LMG 18311, stw: *Streptococcus thermophilus* MN ZLW 002, stn: *Streptococcus thermophilus* ND03. The boxplots for the different strains of the same species are shown in the same color. See Figure 6 for the plot that shows the ratio of genes with antisense transcription (instead of ratio of antisense reads).
