# Supplementary material for: Strand-specific community RNA-seq reveals prevalent and dynamic antisense transcription in human gut microbiota
Source: Front Microbiol. 2015 Sep 1;6:896. doi: 10.3389/fmicb.2015.00896 (PMC4555090; doi:10.3389/fmicb.2015.00896)
Supplement: Supplementary file 5 [file Image_2.PDF]

This document contains Supplementary Figure 2.

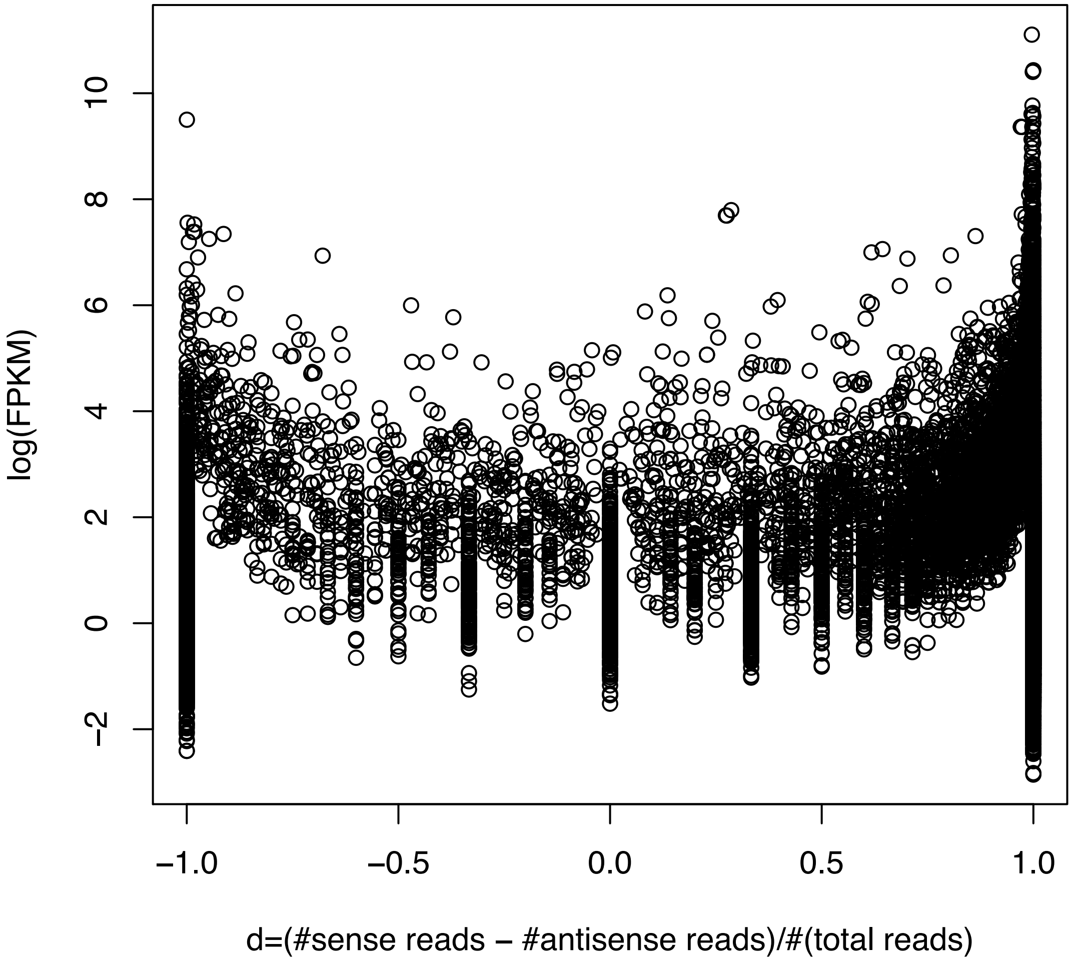

Supplementary Figure 2. A plot of the gene expression (*log*(FPKM)) versus *d,* using RNA-seq data from individual 1 (X310763260), using all genes with RNA-seq support from 47 strains. Each circle represents a gene. The y-axis shows the gene expression (*log*(FPKM)) and the x-axis shows *d,* which is close to 1 for genes with mostly sense transcription, and -1 with mostly antisense transcription. A total of 30,493 genes are shown in this plot. See Figure 7 for the plot using a subset of the genes.
